# Quantitation and integrity evaluation of RNA genome in lentiviral vectors by direct reverse transcription-droplet digital PCR (direct RT-ddPCR)

**DOI:** 10.1038/s41598-023-41644-x

**Published:** 2023-09-02

**Authors:** Zhiyong He, Edward J. Kwee, Megan H. Cleveland, Kenneth D. Cole, Sheng Lin-Gibson, Hua-Jun He

**Affiliations:** Material Measurement Laboratory, National Institute of Standards and Technology, 100 Bureau Drive, MS 8312, Gaithersburg, MD 20899 USA

**Keywords:** Biological techniques, Cancer, Biomarkers, Molecular medicine, Oncology

## Abstract

Lentiviral vectors (LV) have proven to be powerful tools for stable gene delivery in both dividing and non-dividing cells. Approval of these LVs for use in clinical applications has been achieved by improvements in LV design. Critically important characteristics concerning quality control are LV titer quantification and the detection of impurities. However, increasing evidence concerning high variability in titration assays indicates poor harmonization of the methods undertaken to date. In this study, we developed a direct reverse transcription droplet digital PCR (Direct RT-ddPCR) approach without RNA extraction and purification for estimation of LV titer and RNA genome integrity. The RNA genome integrity was assessed by RT-ddPCR assays targeted to four distant regions of the LV genome. Results of the analyses showed that direct RT-ddPCR without RNA extraction and purification performs similarly to RT-ddPCR on purified RNA from 3 different LV samples, in terms of robustness and assay variance. Interestingly, these RNA titer results were comparable to physical titers by p24 antigen ELISA (enzyme-linked immunosorbent assay). Moreover, we confirmed the partial degradation or the incomplete RNA genomes in the prepared 3 LV samples. These results may partially explain the discrepancy of the LV particle titers to functional titers. This work not only demonstrates the feasibility of direct RT-ddPCR in determining LV titers, but also provides a method that can be easily adapted for RNA integrity assessment.

## Introduction

Lentiviral vectors (LVs) belonging to the *Retroviridae* family are spherical to pleomorphic enveloped particles, are approximately (80 to 100) nm in diameter, and their genome possesses two copies of a positive-sense single-stranded RNA inside a conical capsid^1,2^.

LVs have emerged as powerful and versatile gene transfer vectors for ex vivo and in vivo gene therapy as they can stably integrate into the target cell genome, providing long-term transgene expression, and are capable of delivering a large genetic therapeutic cassette (up to 10 kb) into dividing and non-dividing cells^3^.

To date, there are 201 gene therapy clinical trials using LVs, including 86 CAR-T cell trials that utilize LVs to transduce the T cells and 3 CAR-NK cell trials that utilize LVs to transduce the NK cells (ClinicalTrials.gov, accessed on June 1st, 2023). Six LV- based CAR T-cell therapies (Kymriah, Yescarta, Tecartus, Abecma, Breyanzi, and Carvykti) have been approved by the Food and Drug Administration (FDA). In efforts towards delivery of safe and efficacious doses of LV based gene therapy products, the World Health Organization, Lentigen, and researchers in France have independently developed standards and reference materials for vector copy number (VCN) and LV integration sites measurements^4–6^.

Clinical LV preparations are typically produced by transient plasmid-mediated co-expression of viral components in the HEK293T cell line. The production of LVs is therefore a critical step in developing gene therapy as an efficient and cost-effective treatment^7^. To assess process efficiency in terms of yield and recovery, rapid and sensitive quantification methods for LV characterization are highly desired for analyzing crude supernatants and final purified products.

There has been an increase in efforts to develop rapid and robust quantitative characterization methods to measure functional and non-functional lentiviral titer. Currently, many methods for functional and non-functional titer have been described, including HIV-1 p24 antigen enzyme-linked immunosorbent assay (ELISA), RNA vector genome titers, reverse transcriptase activity (RT), dot blot methods, fluorescence-activated cell sorting (FACS) flow cytometry, antibody-based detection on the Virus Counter 3100 (Sartorius, Göttingen, Germany), high-performance liquid chromatography (HPLC), and polymerase chain reaction (PCR)^8–15^. However, there is high variability (100s to 1000s fold differences) in the obtained results, with most of these methods overestimating the functional titer. For instance, in the p24 ELISA, which is only applicable for HIV-1 derived LVs, the protein pool includes a variable amount of free p24 and p24 associated with non-functional vector particles^16^. Non-functional particles are also measured by the RNA titration method. The titer of functional LVs are cell-based methods that mostly done by FACS and PCR-based analysis. FACS analysis is limited to the vectors with expression of fluorescent transgenes and cannot discriminate cells with single or multiple integrations^17^. In addition, results from these cell-based assays are time consuming and complicated, rendering them unsuitable as a routine characterization method for in-process samples.

Rapid and robust quantitation methods for LVs are significant needs in gene therapy. In this study, we developed a direct reverse transcription droplet digital PCR (direct RT-ddPCR) approach without RNA extraction and purification for estimation of LV titer and RNA genome integrity. The RNA genome integrity was assessed by using RT-ddPCR assays against four distant regions of the LV RNA genome. We developed direct, one-step RT-ddPCR assays to reduce both sample handling time and variability. Moreover, we used a lentiviral RNA calibrator to calculate the reverse transcription efficiency of different targets on RNA genome from LV samples.

## Materials and methods

### LV samples

LV samples were purchased from VectorBuilder (Chicago, IL) and Vigene (Rockville, MD). Another LV sample was kindly provided by GSK PLC (Brentford, UK, courtesy of Dr. Conrad Vink). VectorBuilder LVs were designed at National Institute of Standards and Technology (NIST) and packaged at VectorBuilder. The viral particles were aliquoted and stored in Hanks Balanced Salt Solution (HBSS, Thermo Fisher Scientific, Cat# 14025092) buffer at − 80 °C until use. The three LV samples were referred to as samples A, B, and C for analysis.

### Titration by p24 ELISA analysis

A lentiviral p24 ELISA titration kit was purchased from Takara Bio Inc. USA (Lenti-X rapid titer kit, Cat# 632,200). Total p24 was quantified by ELISA following the kit protocol. Free p24 was also quantified by ELISA without adding viral lysis buffer to the reactions. Briefly, 20 µL of viral lysis buffer was added to each well, except for the wells used for free p24 quantification; 200 μL of standard p24 serial dilutions and viral sample serial dilutions were added to each well. The plate was incubated at 37 °C for 1 h. The samples were removed from the wells and the wells were washed with ELISA washing buffer 3 times. A volume of 100 µL of biotin conjugated anti-p24 detector antibody was then added into each well and the plate was incubated at 37 °C for 1 h. The antibodies were aspirated, and the wells were washed with washing buffer 3×. One hundred microliters of Streptavidin-HRP conjugate were added into each well and incubated at room temperature for 30 min. The conjugate was then aspirated, and the wells were washed with washing buffer 3×. One hundred microliters of substrate solution were added into each well and incubated in the dark at room temperature for 30 min. One hundred microliters of stop solution were then added into each well and 450 nm absorption was measured by plate reader (Synergy Mx Microplate Reader, Agilent, Santa Clara, CA). Titer of the lentivirus was calculated based on the assumption of each lentiviral particle containing 2000 molecules of p24^18–20^.

### Viral RNA isolation

Viral RNA isolation was performed using a Qiagen QIAamp viral RNA mini kit (Cat# 52,904, Qiagen, Hilden Germany) by following the kit manufacturer’s protocol. Briefly, 50 µL of lentivirus particles was lysed with 560 µL of Buffer AVL in a microcentrifuge tube with/without 2 × 10^6^ copies of SARS-Cov 2 RNA (a NIST research grade test material RGTM 10169, NIST, Gaithersburg, MD) spike-in, and incubated at room temperature for 10 min. The lysate was then mixed with 560 µL of 100% ethanol. The ethanol and viral lysate mixture was loaded onto a QIAamp Mini column and centrifuged at 6000×*g* for 1 min. The column was then washed with 700 µL wash buffer AW1 once and twice with 500 µL wash buffer AW2. A final spin at 12,000×*g*, for 1 min was performed to remove the residual wash buffer, and the viral RNA was eluted with 50 µL of buffer AVE. The RNA samples were stored at -80 °C until use.

### Viral RNA size analysis

The sizes of isolated viral RNA from lentivirus particles were analyzed on Agilent Bioanalyzer 2000 by using Agilent RNA 6000 Nano Kit (Cat# 5067-1511, Agilent, Santa Clara, CA) and following the instructions of the kit.

### DNA and RNA calibrators synthesis

A DNA template was generated by amplifying most of the lentiviral genome from the GSK lentivirus sample (produced from transduced HEK 293 cells at NIST), using the 5′ primer sequence located at the 5′ long terminal repeat (LTR), and the 3′ primer sequence located at the junction of the woodchuck hepatitis virus post-transcriptional regulatory element (WPRE) and 3′ LTR (forward primer: TAATACGACTCACTATAGGGGTCTCTCTGGTTAGATCT and reverse primer: TGCCTTGTAAGTCATTGGTCTTAAAG). A T7 promoter sequence was added to the 5′ primer sequence. As a result, a 3823 bp DNA fragment (Supplemental Fig. 1A) was amplified and purified with Zymo DNA clean and a concentration kit (Cat# D4033, Zymo Research, irvine, CA) (Supplemental Fig. 1B). Utilizing the T7 promoter in the amplified GSK lentiviral DNA as template, lentiviral RNA was in vitro synthesized by using HiScribe T7 Quick High Yield RNA Synthesis kit (New England Biolabs, Cat# E2050S). In vitro synthesized lentiviral RNA was purified with Qiagen RNeasy kit (Qiagen Cat# 74134) according to the kit protocol and then confirmed the right size with a Bioanalyzer 2100 (Agilent, Santa Clara, CA) (Supplemental Fig. 1C). One nanogram of synthetic lentiviral RNA is 4.86 × 10^8^ copies based on the calculated molecular weight.

### One-step reverse transcription-droplet digital PCR (RT-ddPCR) and one-step direct RT-ddPCR

One-step RT-ddPCR experiments were performed to measure the lentiviral RNA genome copy numbers by using Bio-Rad One-step RT-ddPCR advanced kit for probes (Bio-Rad, Cat# 1864022). Briefly, eluted RNA was serially diluted in RNA storage solution (RSS) until reaching the detection range of ddPCR, and then combined with reagents from the One-Step RT-ddPCR Kit and two primer/probe sets each with final primer concentrations of 900 nmol/L and probe concentrations of 50 nmol/L. To measure viral RNA genome copies, the ddPCR assay target 5′ LTR^6^, Psi^6^, RRE, GFP and WPRE were used. The primer and probe sequences are listed in Table 1. After the RT-ddPCR reaction setup, droplets were generated by the Bio-Rad manual Droplet Generator according to the manufacturer’s instructions. Then the droplets were subjected to endpoint PCR thermal cycling with the following protocol: 1 cycle of 50 °C for 1 h; 1 cycle of 95 °C for 10 min; 40 cycles of 94 °C for 30 s, 60 °C for 1 min; and 1 cycle of 98 °C for 10 min followed by a 4 °C hold. Droplet results were acquired by QX200 Droplet Reader and the ddPCR data was analyzed by QuantaSoft 1.7.4 or QX Manager 1.2 software.

**Table 1:** Primer and probe information for ddPCR assays.

Primer/probe name^a^	DNA sequence	Amplicon size
5′ LTR-F^6^	TGCCCGTCTGTTGTGTGACT	140
5′ LTR-R^6^	CGAGTCCTGCGTCGAGAGA	
5′ LTR-P	Fam-AGTCCCTGTTCGGGC-MGB	
Psi-F^6^	CTCTCTCGACGCAGGACTCG	126
Psi-R^6^	GACGCTCTCGCACCCATCT	
Psi-P	VIC-CTCTTGCCGTGCGCG-MGB	
RRE-F	AAACTCATTTGCACCACTGC	106
RRE-R	AATTTCTCTGTCCCACTCCATC	
RRE-P	Fam-TGTGCCTTGGAATGC-MGB	
eGFP-F	TGCTGCCCGACAACCACT	80
eGFP-R	CAGGACCATGTGATCGCG	
eGFP-P	Fam-TGAGCAAAGACCCCAAC-MGB	
WPRE-F	TTACGCTATGTGGATACGCTG	109
WPRE-R	TCATAAAGAGACAGCAACCAGG	
WPRE-P	VIC-AGGAGAAAATGAAAGCCATAC-MGB	

^a^F, forward primer; R, reverse primer; P, probe.

One-step direct RT-ddPCR experiments were performed to measure the lentiviral RNA genome copy numbers by using similar protocol as One-step RT-ddPCR without RNA extraction from the viral particles. Treated or untreated lentiviral particles instead of diluted RNA were directly applied to the RT-ddPCR reaction mixtures. Lentiviral particles were heat treated at different temperatures from (56 to 95) °C for (10 or 15) min (Fig. 4). Untreated lentiviral particles were used as control. Between 3000 and 10,000 lentiviral particles were added to each ddPCR reaction.

### Statistical analysis

Statistical analysis was carried out by using GraphPad Prism version 9.4.1 software or Microsoft Excel. All data presented here is mean ± standard deviation. *P* values less than 0.05 was considered as significant between the samples compared.

### Ethical approval

Certain commercial equipment, instruments, and materials are identified to specify the experimental procedure. In no case does such identification imply recommendation or endorsement by the National Institute of Standards and Technology, nor does it imply that the materials or equipment are necessarily the best available for the purpose.

## Results

### Lentivirus titration by p24 ELISA

All lentivirus samples were titrated by p24 ELISA to confirm the physical titer. The lentivirus particles were diluted in PBS with 10% FBS by serial dilution. After determining the dilution range within the ELISA kit linear detection range, 3 serial dilutions of each sample were used for titration. All 3 dilutions resulted in similar p24 titer (data not shown). Interestingly, the p24 ELISA titration method from our experiments showed different titers compare with other titration methods (Table 2). The discrepancy could be a result of different lentivirus purification methods that lead to the different purity of the final lentivirus or different p24 standards used. Free p24 protein was also measured in the lentivirus sample without the lysing process. Because the p24 protein is covered by viral envelope, only free p24 protein can be detected by ELISA when the lentivirus is intact. ELISA results showed that about 2% of p24 protein is free p24 (free p24: 2.8 × 10^9^ ± 1.5 × 10^8^, total p24: 1.4 × 10^11^ ± 1.1 × 10^10^). Calculated from the molecular weight of p24, 1 pg of p24 is equivalent to 2.5 × 10^7^ molecules. Each lentivirus contains about 2000 p24 molecules, therefore, 1 pg of p24 is about 1.25 × 10^4^ lentivirus particles. The equation for lentivirus titer calculation is: p24 amount (pg) × 1.25 × 10^4^ × dilution factor.

**Table 2:** Comparison of titers resulted from different titration methods (data presented mean ± SD).

Lentivirus samples	Manufacturer’s titer	p24 ELISA titer	RNA extraction ddPCR titer*	Direct RT-ddPCR titer*	Transduction titer (HEK293)*
A	4.46 × 10^8^	2.27 × 10^11^ ± 5.82 × 10^10^	1.35 × 10^11^ ± 1.30 × 10^8^	1.05 × 10^11^ ± 3.61 × 10^9^	9.94 × 10^8^ ± 9.00 × 10^6^
B	7.30 × 10^8^	5.78 × 10^10^ ± 5.85 × 10^9^	1.56 × 10^10^ ± 2.72 × 10^9^	1.69 × 10^10^ ± 8.91 × 10^8^	Not tested
C	1.76 × 10^9^	2.12 × 10^10^ ± 4.94 × 10^9^	9.29 × 10^9^ ± 2.14 × 10^8^	1.80 × 10^10^ ± 1.53 × 10^8^	Not tested

*The titers were determined by RRE assay.

### Viral RNA extraction efficiency

**Figure 1:**
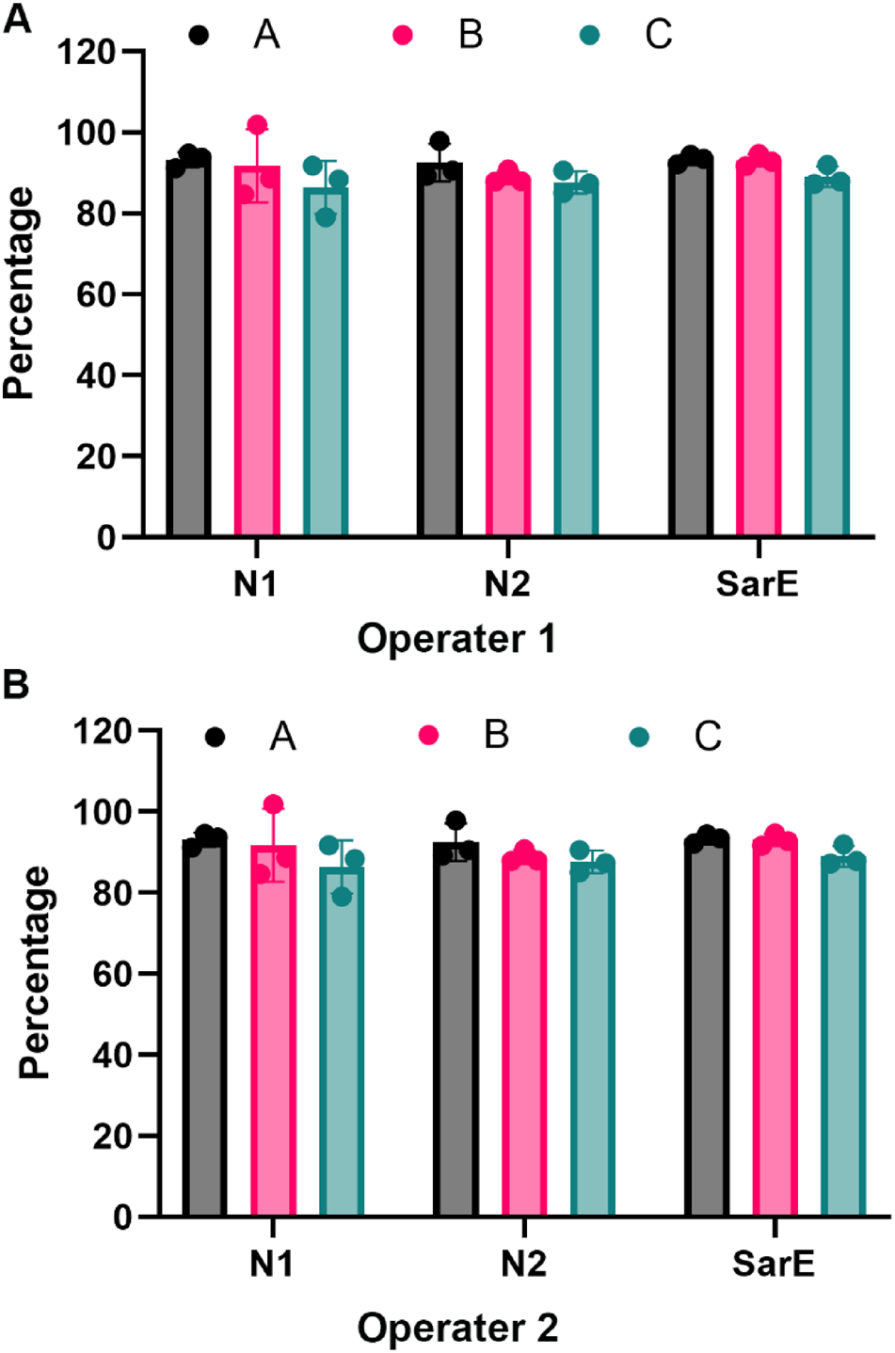
RNA recovery rates from 2 independent operators calculated by spike-in RNA. In each LV sample (A, B, and C), 2 × 10^6^ copies of RGTM 10169 were spiked in after viral particles were lysed with lysis buffer. The RNA recover rates were calculated by percentage of detected copy numbers of each detecting target within the RGTM 10169 RNA out of total spiked in copy numbers. Bars present mean ± SD, n = 3.

To determine the lentiviral RNA extraction efficiency, SARS-CoV-2 RNA Fragment 1, a NIST research grade test material (RGTM 10169) was used as a calibrator. After the lentivirus particles were lysed in the lysis buffer, 2 × 10^6^ copies of RGTM 10169 were spiked into the lysis mixture followed by viral RNA extraction as described above. The RNA samples were serially diluted and used as a template for one-step RT-ddPCR. Three assays were used to detect the copy numbers of RGTM 10169 in the direct RT-ddPCR. RNA recovery rate was calculated as the percentage of detected copy numbers versus total spiked in copy numbers. As shown in Fig. 1, the RGTM 10169 RNA recovery rate determined by all 3 assays (Targeting two independent regions of the SARS-CoV-2 nucleocapsid gene (N1, N2) and envelope gene E-Sarbeco (SarE)) was between 86 and 93%. Interestingly, operators 1 and 2 in our lab obtained very similar RNA recovery rate. Sample A showed the highest RNA extraction efficiency (93%) and Sample C showed a relative lower recovery rate (86–89) % (Fig. 1). The extraction efficiency of the spiked-in RGTM 10169 RNA was used to evaluate the recovery rate of released LV RNA genome from the lentiviral particles. The pre-extraction LV RNA genome copy concentration was calculated by using the detected post-extraction LV RNA genome copy concentration normalized to the extraction efficiency of RGTM.

### Establish one-step direct RT-ddPCR using different primers/probe sets to titrate lentivirus RNA genome

**Figure 2:**
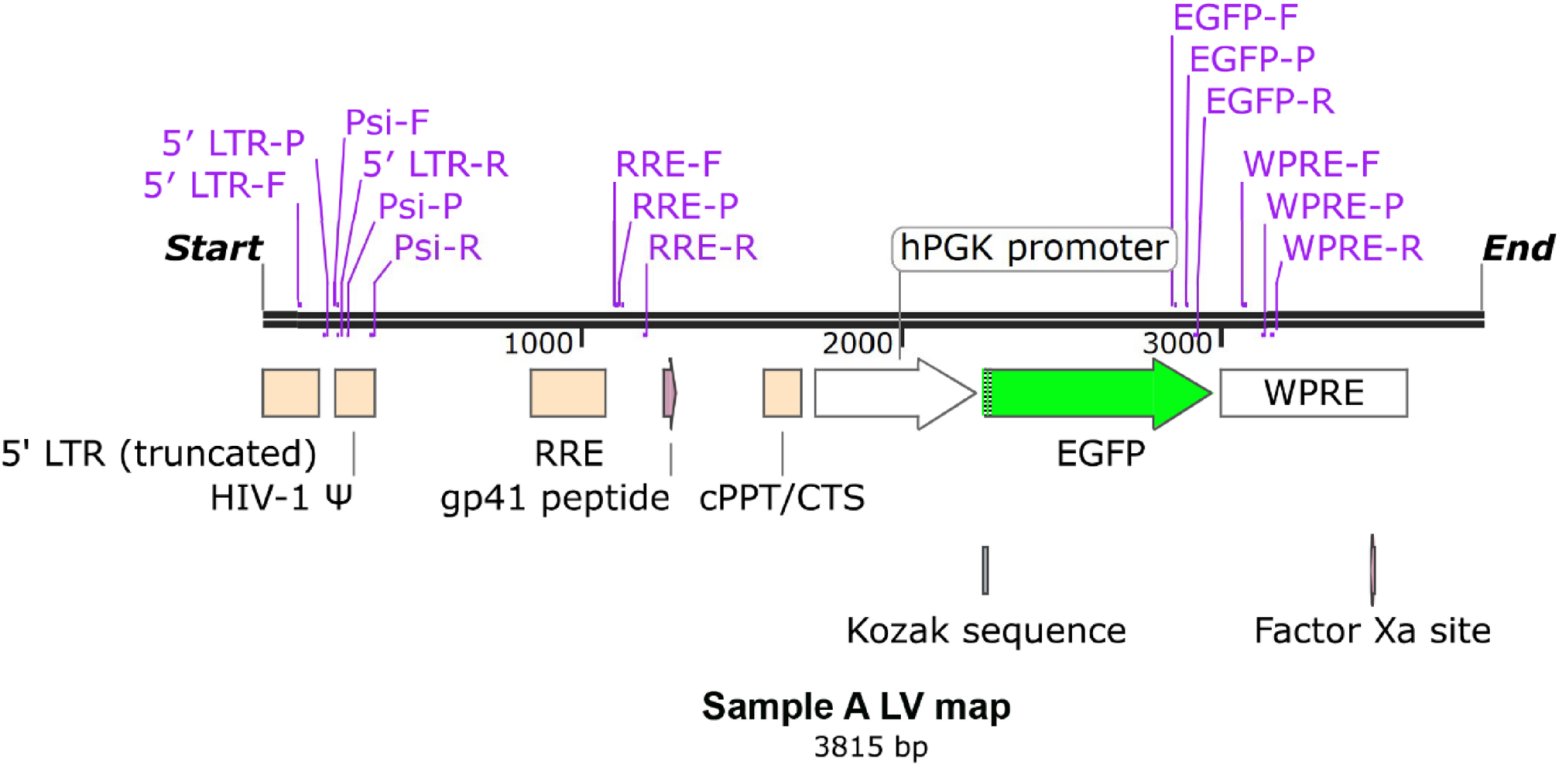
Schematic diagram showing the locations five sets of ddPCR primers/probe for LV genome quantitation by using a Sample A LV sequence as an example.

**Figure 3:**
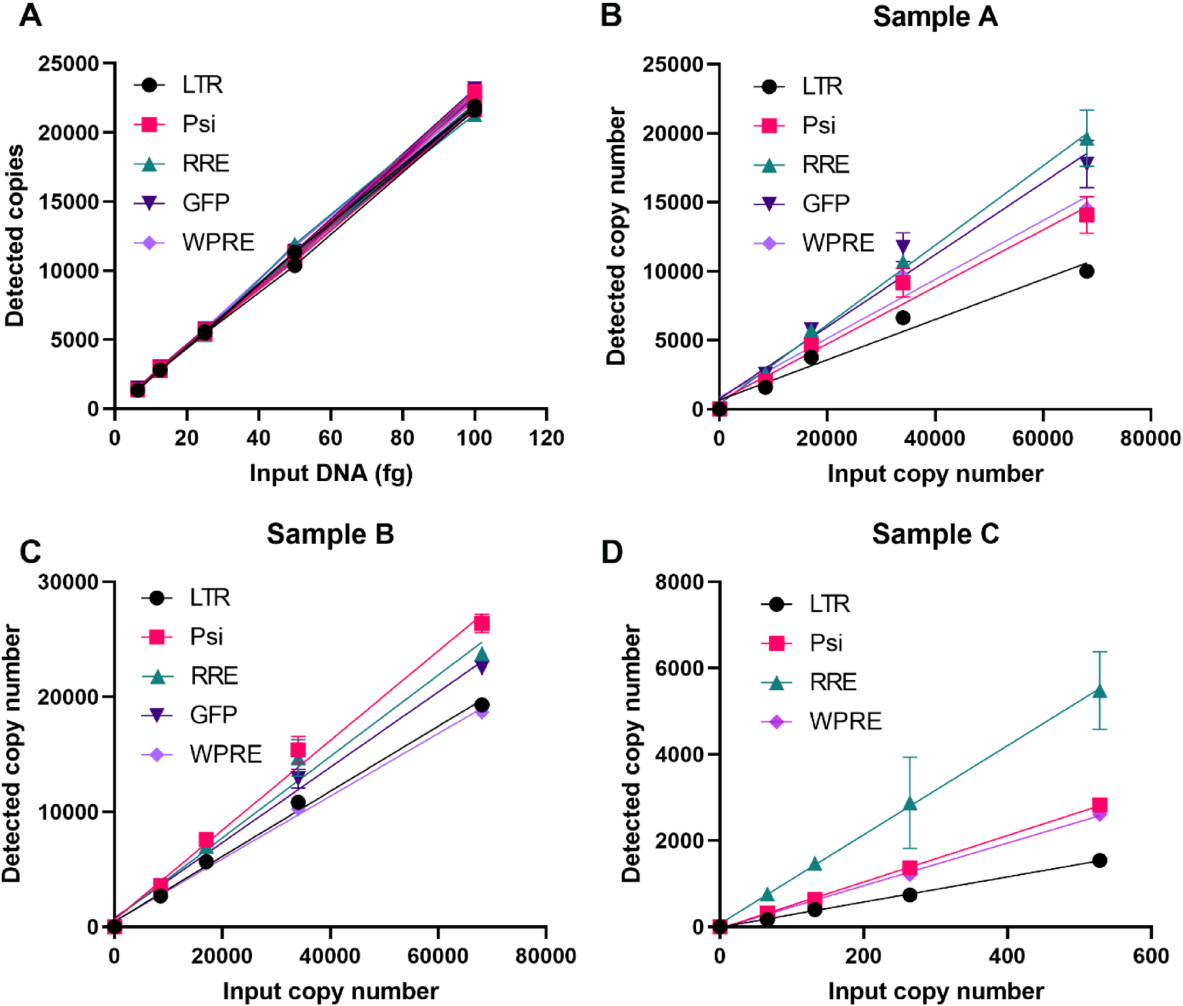
Using 5 primers/probe sets to titrate lentivirus by detecting RNA genome copy number. (**A**) By using lentivirus genome DNA as template, 5 sets of primers/probe were all able to detect the viral genome DNA and showed equivalent PCR efficiency. (**B**–**D**) All 5 sets of primers/probe detected lentivirus RNA genome in one-step direct RT-ddPCR reactions. Sample C does not contain GFP gene, therefore there were only 4 assays displayed in Fig. 3D. Input copy number was determined by viral titer provided by the companies. All data presents mean ± SD, n = 3.

Lentiviral RNA preparation is a step that may cause variations for lentivirus particles titration. One-step direct RT-ddPCR could reduce the sample handling time and the variability introduced by RNA isolation. Five sets of ddPCR assays were designed and used to titrate the LV genome, as shown in Fig. 2 by using a GSK third generation of LV as an example to illustrate the genes and primer locations. We first used a lentivirus DNA sequence as a template to validate the primers/probe assays. As shown in Fig. 3A, all 5 sets of primers/probe can detect lentiviral DNA genome at the same detection level in different dilutions, indicating that the primer/probe sets are equally efficient in amplifying lentiviral DNA. The linear correlation of each primers/probe set was excellent, with R^2^ > 0.997. We then tested 5 sets of primers/probe for titrating lentiviral RNA genome in the direct RT-ddPCR reactions. The LV samples were serially diluted and used for direct RT-ddPCR without any treatment. All 5 sets of primers/probe detected lentiviral RNA genome without RNA extraction (Fig. 3B,C,D). All 5 sets of primers/probe had near perfect linearity with different dilution of lentivirus in all 3 sources (Fig. 3B,C,D). Interestingly, the highest titer obtained by RRE primers/probe and p24 ELISA titer are within the same order of magnitude.

### Heat inactivation of lentivirus particles increased one-step RT-ddPCR detection sensitivity

**Figure 4:**
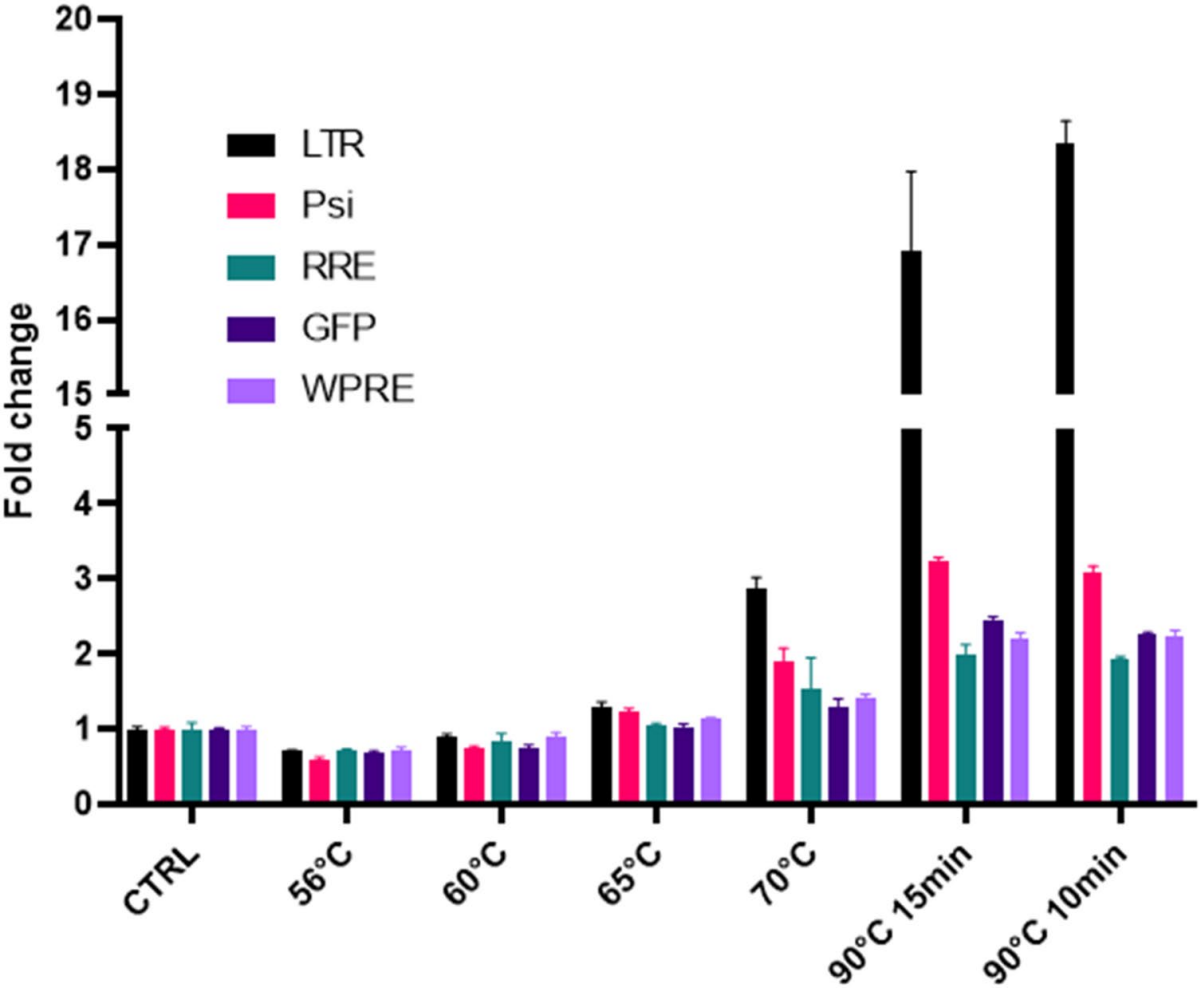
Optimization of one-step direct RT-ddPCR reaction. Lentivirus particles were treated at different temperatures for 10 min to 15 min before one-step direct RT-ddPCR reaction. Bars present mean ± SD, n = 3.

We showed that the primers/probe sets can detect lentiviral RNA genome without RNA extraction (Fig. 3B,C,D). Because interaction between the viral RNA genome and capsid protein could interfere with direct digital PCR efficiency, we hypothesized that heat inactivation of the lentivirus can increase the detection level of lentiviral RNA. To test this hypothesis, we treated the lentivirus particles at 56 °C, 60 °C, 65 °C, 70 °C, and 90 °C for 15 min. We found that low temperature treatment did not increase the detection level (56 °C and 60 °C). But 90 °C heat treatment of the lentivirus increased the detection of 5′ LTR more than 17-fold comparing with the reactions from the lentivirus that were not treated (Fig. 4). The detection of the other 4 assays also increased two to four- fold comparing with samples without heat treatment (Fig. 4). We further compared the detection levels of 90 °C treatment for 10 min and 15 min. A slightly higher detection level was found after 10 min of treatment (Fig. 4). A comparison between 90 and 95 °C heat treatment did not show a significant difference in detection level of any primer/probe set (Supplemental Fig. S2). Therefore, we used 90 °C, 10 min heat inactivation for the rest of direct RT-ddPCR assays.

### Effect of diluents on RT-ddPCR detection level

**Figure 5:**
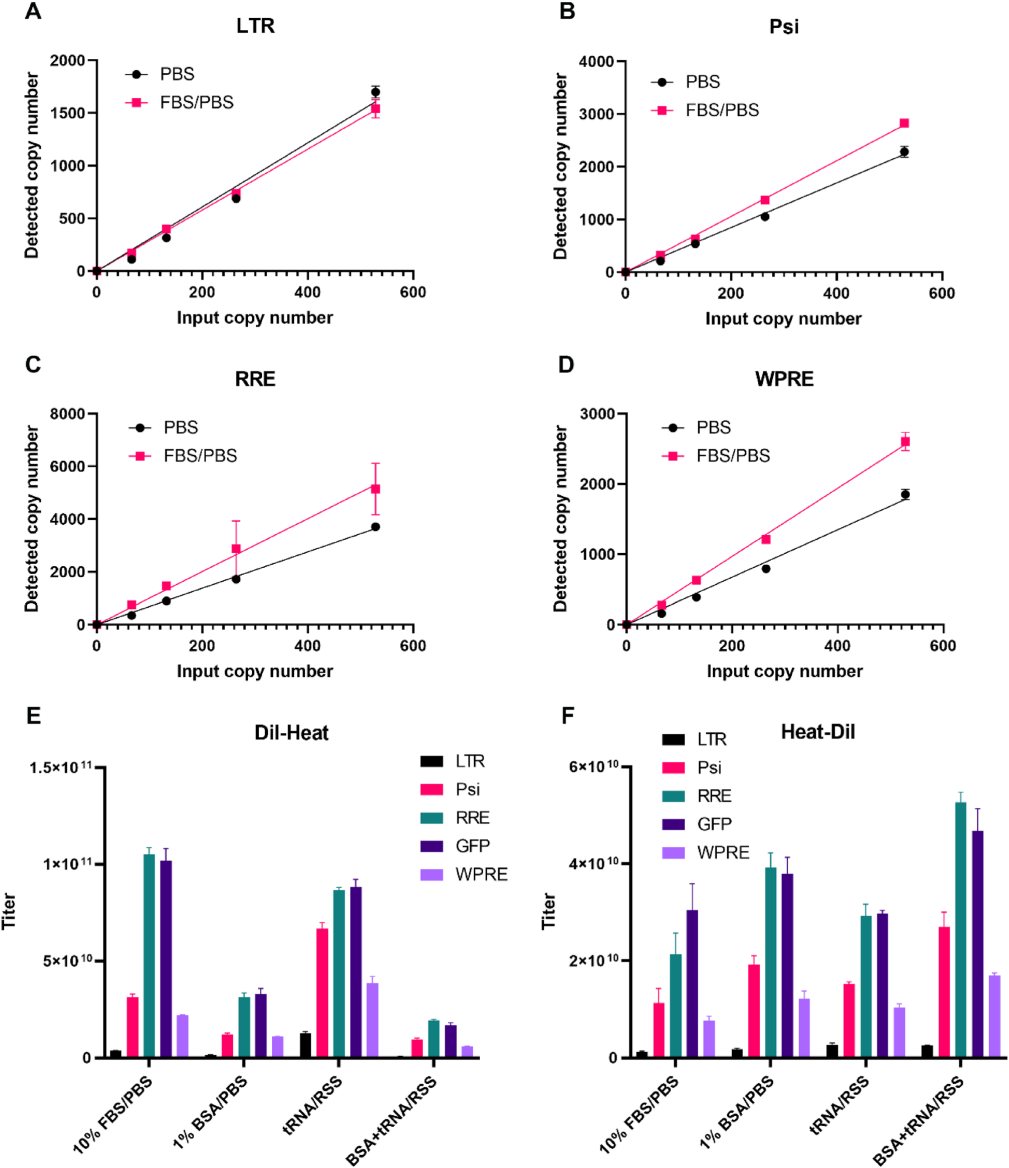
Effect of diluent on the one-step RT-ddPCR detection. (**A**) Comparison of LTR one-step RT-ddPCR results from the LV diluted in PBS and 10% FBS/PBS. (**B**) Comparison of WPRE one-step RT-ddPCR results from the LV diluted in PBS and 10% FBS/PBS. (**C**) Comparison of Psi one-step RT-ddPCR results from the LV diluted in PBS and 10% FBS/PBS. (**D**) Comparison of LTR one-step RT-ddPCR results from the LV diluted in PBS and 10% FBS/PBS. (**E**) Comparison of one-step RT-ddPCR results of 5 assays from the LV diluted in different diluents before heat treatment. (**F**) comparison of one-step RT-ddPCR results of 5 assays from the LV diluted in different diluents after heat treatment. All data presents mean ± SD, n = 3.

We then investigated whether the diluents used to dilute the lentivirus affect RT-ddPCR detection level. Our first hypothesis was that adding proteins such as fetal bovine serum (FBS) to PBS will reduce lentivirus particles binding to the tube wall and increase the RT-ddPCR detection level. To test this hypothesis, we performed serial dilution of Sample C in PBS and PBS containing 10% FBS before heat inactivation and RT-ddPCR. As shown in Fig. 5A–D, there was no difference of LTR detection level at higher concentrations, and a 25% to 52% increase in detection at the lower concentration. For the other 3 assays (Psi, RRE, and WPRE), adding 10% FBS to PBS all increased detection level (39–117%) as shown in Figure. We next tested whether other diluent such as RSS would protect RNA from degradation after RNA releasing by heat inactivation. The lentivirus sample A was treated in 2 different protocols: (a) serially diluted into different diluents then the final diluted sample was heat inactivated at 90 °C for 10 min; or (b) first diluted 10× in different diluents, then heat inactivated at 90 °C for 10 min and then further diluted in RSS containing 20 ng/µL yeast tRNA (tRNA/RSS) to the targeted dilution for RT-ddPCR. As shown in Fig. 5E,F, diluting the lentivirus particles in the diluents before heat inactivation helped prevent the RNA degradation in the solutions. These results also indicated that dilution of the lentivirus samples in tRNA/RSS before heat inactivation resulted in the highest detection level for LTR, Psi, and WPRE amplicons.

### Measure the integrity of lentiviral RNA genome by one step direct RT-ddPCR

**Figure 6:**
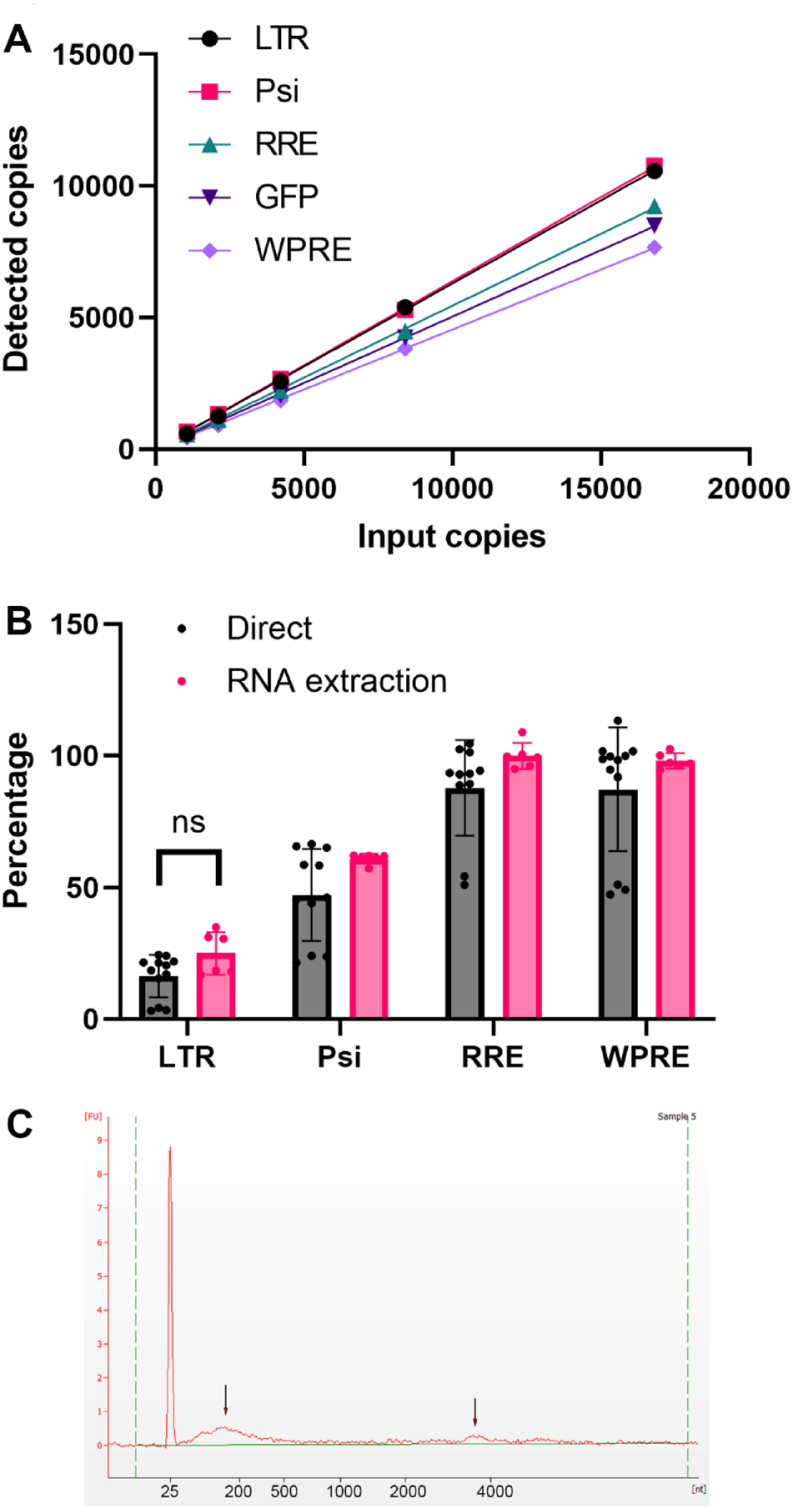
LV RNA genome integrity. (**A**) RT efficiency of 5 primer/probe sets assays. All data presents mean ± SD, n = 3. (**B**) Comparison of LV RNA genome integrity determined by direct RT-ddPCR and by RT-PCR on extracted RNA using in vitro transcribed RNA as calibrator. Average of 4 experiments, bars present mean ± SD. (**C**) RNA size analysis by Bioanalyzer on RNA extracted from Sample A.

Within the lentivirus particles, there could be empty particles without RNA genome, truncated RNA genome, or intact RNA genome. To measure the lentiviral RNA genome integrity, we first synthesized a GSK lentiviral RNA genome in vitro as calibrator. Dilution linearity and reverse transcription efficiency (RT efficiency) was analyzed in one-step RT-ddPCR and the results are shown in Fig. 6A and Supplemental Fig. S3. All 5 primers/probe sets had very good dilution linearity and the RT efficiency varied from 39.7 to 62.4% (Fig. 6A and Supplemental Fig. S3, Table S1).

We utilized the synthesized lentiviral RNA (derived from the GSK sample) as a calibrator to measure the integrity of RNA that was packed inside the lentivirus particles. The detected copy numbers of each element from lentiviral RNA genome were normalized to its RT efficiency, and the element with the highest detection level was assigned as 100% (Fig. 3A and Table S1). For most cases, RRE had the highest detection level. The highest detected element was assigned as 100% for each tested lentivirus and other elements were normalized to the detection level of the highest element. Theoretically, identical detection levels of all elements will be achieved for a LV with full RNA genome after RT efficiency normalization. As shown in Fig. 6B, 5′-LTR was the lowest detectable element, which is about 16.34% ± 8.07% for Sample A (Average results of 4 experiments performed on the same batch of Sample A), 21.83% ± 0.63% for Sample B (data not shown), and 15.94% ± 0.48% for Sample C (data not shown). The percentage of LTR to the highest detected element did not show statistic difference between direct one step RT-ddPCR and RT-ddPCR on RNA extracted from LV particles (Fig. 6B). We further confirmed the RNA sizes on Bioanalyzer by using Agilent RNA 6000 nano kit. The intact sample A lentiviral RNA genome is around 4000 nt. As shown in Fig. 6C, the majority of the RNA isolated from the lentivirus particles is at 25–500 nt sizes. There is only a small amount of RNA showed around 4000 nt. This data indicated that most of the lentivirus particles had truncated RNA genomes, especially in the 5′-LTR region.

### Comparison of one step direct RT-ddPCR and RNA extraction RT-ddPCR method

**Figure 7:**
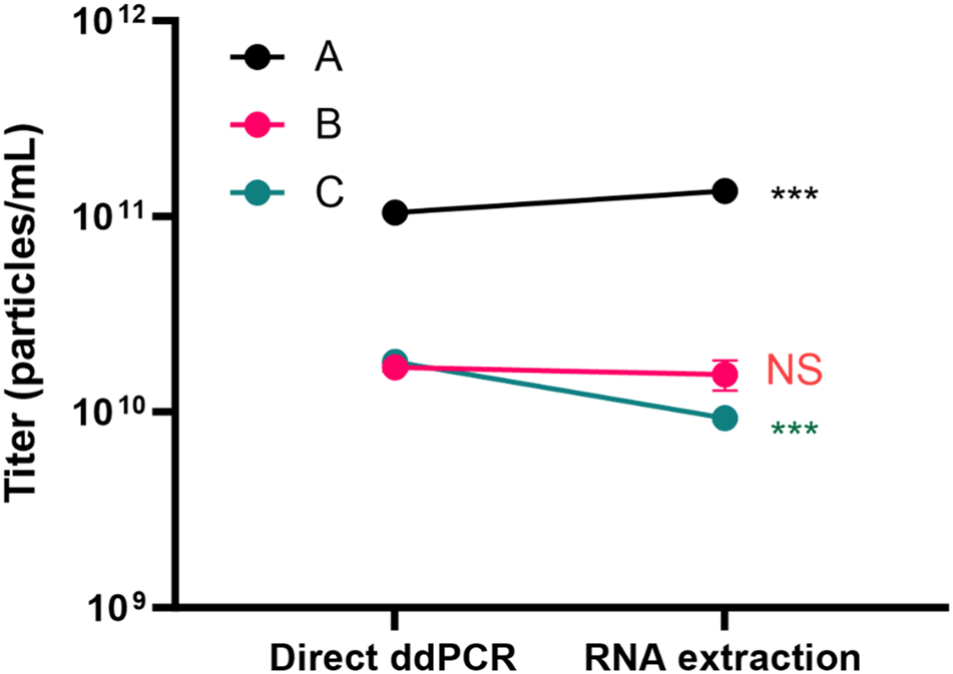
Titer comparison between one step direct RT-ddPCR and RNA extracted from LV using RRE primer/probe set. Direct ddPCR indicates one step direct RT-ddPCR (Data from all three samples). ***indicates *P* < 0.001; NS, indicates not significant.

We compared the titer units detected by these RT-ddPCR with and without RNA extraction from viral particles. Interestingly, the RRE primer/probe set had similar titers detected by both methods. For sample A, we detected, we detected 1.05 × 10^11^ ± 3.61 × 10^9^ from one step direct RT-ddPCR and 1.35 × 10^11^ ± 1.30 × 10^8^ from RNA extraction sample (Fig. 7 and Table 2). Similarly, we detected 1.69 × 10^10^ ± 8.19 × 10^8^ and 1.56 × 10^10^ ± 2.72 × 10^9^ from Sample B, respectively. Slightly higher level of detection was observed from Sample C by one step direct RT-ddPCR at 1.80 × 10^10^ ± 1.53 × 10^8^ comparing to 9.29 × 10^9^ ± 2.14 × 10^8^ by RNA extraction RT-ddPCR method (Fig. 7, Table 2).

## Discussion

The increasing application of LVs in gene transfer and/or gene therapy demands a rapid and robust measurement of LV titers and quality. There are a few existing LV titration methods, such as p24 ELISA, RNA extraction followed by qPCR, and cell-based assays. P24 ELISA measures the amount of LV capsid protein p24 and assumes that each LV particle has 2000 molecules of p24^18–20^. Therefore, this method has multiple limitations. First, the ELISA p24 standard can differ from company to company and from batch to batch. Second, the assay cannot discriminate between associated p24 and free p24. Here we demonstrated that we detected approximately 2% of free p24 in the final LV product. Third, the assay cannot distinguish empty, partial, or truncated LV particles. PCR based assays quantify the copy number of LV RNA genome. However, RNA is relatively unstable once extracted from LV particles. RNA extraction and handling can introduce more variability to the measurements as a result of RNA extraction kits and operator differences. In this study, we developed one-step direct RT-ddPCR method to quantify the LV RNA genome copy numbers. This one-step RT-ddPCR method measures the copy numbers of 4 common elements of lentivirus RNA genome and eGFP (if the LV genome contains eGFP gene). It can reduce the RNA handling time and variability from the RNA extraction process.

When the lentivirus particles are packaged within the host cells, the lentivirus particles can contain partial RNA genome, no RNA genome (empty virus particles), or an intact RNA genome. Most of currently used methods to titrate the LVs are not able to determine the integrity of the LV RNA genome. In this study, we developed a method to measure the integrity of LV RNA genome. By using an RNA calibrator, we were able to determine the RT efficiency of each ddPCR target element and determine the percentage of lentiviral particles that have intact RNA genomes (Fig. 6). Capsid protein p24 titer cannot predict the transduction titer. As shown in in Table 2, Sample A transduction titer provided by the manufacturer was 4.46 × 10^8^/mL, whereas p24 titer was 1.9 × 10^11^, which was approximately 100-fold higher. By using 5 different ddPCR targets and the RNA calibrator, we detected that about 16.34% of the RNA genome in Sample A was intact. The ddPCR targeting LTR showed the lowest titer among the 5 PCR targets (4.31 × 10^9^ ± 7.12 × 10^8^) and it was close to the transduction titer we determined by using HEK293 cells (9.94 × 10^8^ ± 9.0 × 10^6^). Lentivirus transduction efficiency varies based on the transduction protocol, cell type, cell quality and other factors. Taken together, this one-step direct RT-ddPCR method provides a rapid and robust measurement of LV titers and quality.

### Supplementary Information

Supplementary Information.

## Data Availability

The datasets generated during and/or analyzed during the current study are available from the corresponding author(s) on reasonable request.

## References

[1] Durand S, Cimarelli A (2011). The inside out of lentiviral vectors. Viruses.

[2] Tolmachov OE, Tolmachova T, Al-Allaf FA (2011). Designing lentiviral gene vectors. Viral Gene Therapy.

[3] Escors D, Breckpot K (2010). Lentiviral vectors in gene therapy: Their current status and future potential. Arch. Immunol. Ther. Exp. (Warsz.).

[4] Corre G (2022). Lentiviral standards to determine the sensitivity of assays that quantify lentiviral vector copy numbers and genomic insertion sites in cells. Gene Ther..

[5] Paugh BS (2021). Reference standards for accurate validation and optimization of assays that determine integrated lentiviral vector copy number in transduced cells. Sci. Rep..

[6] Zhao Y, Stepto H, Schneider CK (2017). Development of the first world health organization lentiviral vector standard: Toward the production control and standardization of lentivirus-based gene therapy products. Hum. Gene Ther. Methods.

[7] Ferreira CB (2020). Lentiviral vector production titer is not limited in HEK293T by induced intracellular innate immunity. Mol. Ther. Methods Clin. Dev..

[8] Barczak W, Suchorska W, Rubis B, Kulcenty K (2015). Universal real-time PCR-based assay for lentiviral titration. Mol. Biotechnol..

[9] Delenda C, Gaillard C (2005). Real-time quantitative PCR for the design of lentiviral vector analytical assays. Gene Ther..

[10] Geraerts M, Willems S, Baekelandt V, Debyser Z, Gijsbers R (2006). Comparison of lentiviral vector titration methods. BMC Biotechnol..

[11] Lizee G (2003). Real-time quantitative reverse transcriptase-polymerase chain reaction as a method for determining lentiviral vector titers and measuring transgene expression. Hum. Gene Ther..

[12] Sastry L, Johnson T, Hobson MJ, Smucker B, Cornetta K (2002). Titering lentiviral vectors: Comparison of DNA, RNA and marker expression methods. Gene Ther..

[13] Scherr M, Battmer K, Blömer U, Ganser A, Grez M (2001). Quantitative determination of lentiviral vector particle numbers by real-time PCR. BioTechniques.

[14] Transfiguracion J (2020). Rapid in-process monitoring of lentiviral vector particles by high-performance liquid chromatography. Mol. Ther. Methods Clin. Dev..

[15] Wang Y, Bergelson S, Feschenko M (2018). Determination of lentiviral infectious titer by a novel droplet digital PCR method. Hum. Gene Ther. Methods.

[16] Nair A (2008). A rapid and efficient branched DNA hybridization assay to titer lentiviral vectors. J. Virol. Methods.

[17] Ding B, Kilpatrick DL (2013). Lentiviral vector production, titration, and transduction of primary neurons. Methods Mol. Biol..

[18] Layne SP (1992). Factors underlying spontaneous inactivation and susceptibility to neutralization of human immunodeficiency virus. Virology.

[19] Arthur LO (1992). Cellular proteins bound to immunodeficiency viruses: Implications for pathogenesis and vaccines. Science.

[20] Tang S (2010). Characterization of immune responses to capsid protein p24 of human immunodeficiency virus type 1 and implications for detection. Clin. Vaccine Immunol..

