# Supplementary material for: Quantitation and integrity evaluation of RNA genome in lentiviral vectors by direct reverse transcription-droplet digital PCR (direct RT-ddPCR)
Source: Sci Rep. 2023 Sep 2;13:14470. doi: 10.1038/s41598-023-41644-x (PMC10475045; doi:10.1038/s41598-023-41644-x)
Supplement: Supplementary file 1 — Supplementary Information. [file 41598_2023_41644_MOESM1_ESM.docx]

**Supporting Information**

**Quantitation and Integrity Evaluation of RNA Genome in Lentiviral Vectors by Direct Reverse Transcription-Droplet Digital PCR (RT-ddPCR)**

Zhiyong He, Edward J Kwee, Megan H. Cleveland, Kenneth D. Cole, Sheng Lin-Gibson, Hua-Jun He

1. pages

1. **Synthesis of DNA and RNA Calibrators**

A DNA template/calibrator was generated by amplifying the lentiviral genome from GSK lentivirus transduced sample (produced from transduced HEK 293 cells in NIST lab), using the 5’ primer sequence located at 5’ LTR, and the 3’ primer sequence located at the junction of WPRE and 3’ LTR. A T7 promoter sequence was added to the 5’ primer sequence as shown in the Figure S1 (A). The amplified DNA size was verified (B) and purified, lentiviral RNA calibrator was *in vitro* synthesized by using the purified DNA as template and then characterized by Bioanalyzer (C).

Figure S1. Synthesis of GSK LV DNA and RNA calibrators. Schematic diagram shows the GSK LV primers and elements (A); flash gel for the DNA template size analysis (B); and Bioanalyzer analysis of the purified *in vitro* synthesized RNA calibrator (C).

2. **Optimization of heat inactivation for direct RT-ddPCR titration of lentivirus RNA genome**

One-step direct RT-ddPCR experiments were performed to measure the lentiviral RNA genome copy numbers. Lentivirus Sample A was heated at temperature 90 or 95 ˚C for 5, 10, 15 or 20 min and then detected by direct RT-PCR measurement. Four ddPCR assays for 5’LTR, Psi, RRE and WPRE were used to optimize the heat inactivation. Around 3,000-10,000 lentiviral particles were loaded to each ddPCR reaction.


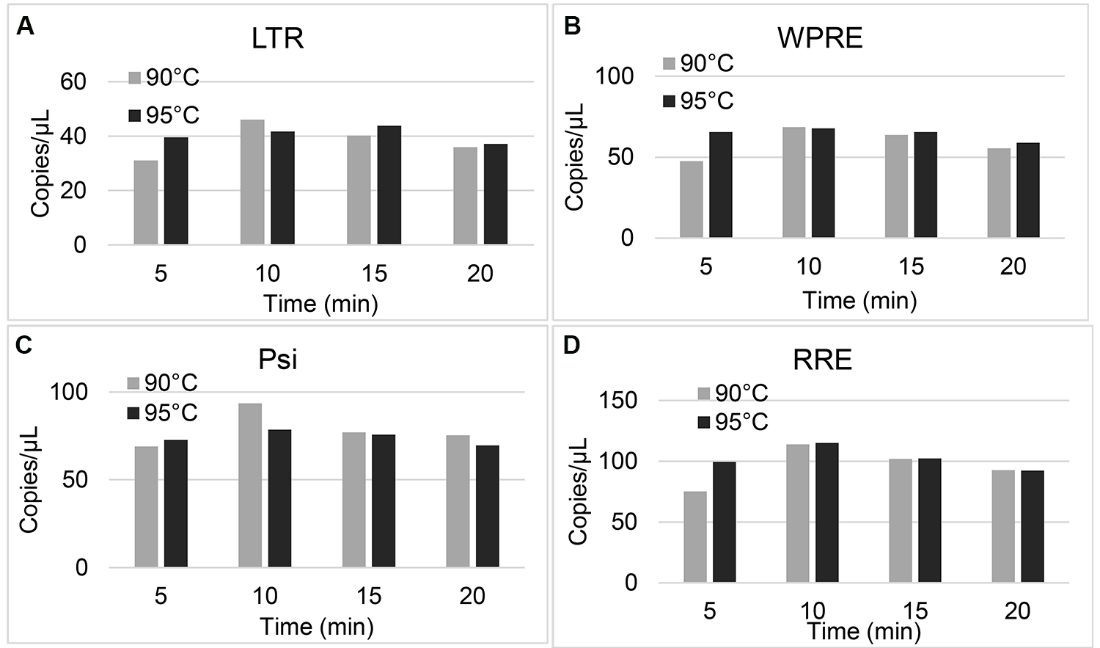


Figure S2. Optimization of heat inactivation for direct RT-ddPCR titration of lentivirus RNA genome.

3. **Reverse transcription efficiency determination by using an *in vitro* synthesized RNA calibrator**

We have shown that 5 ddPCR assays (5’LTR, Psi, RRE, eGFP and WPRE) are equally efficient in amplifying lentiviral DNA in Fig. 3A. The RT efficiency of 5 assays can be determined by RT-ddPCR using a purified *in vitro* synthesized RNA as calibrator. Thus, the lentiviral RNA genome integrity can be evaluated by the RT-ddPCR assays’ results normalized to their RT efficiency.

| Assay target | RT efficiency |
| --- | --- |
| LTR | 62.41% |
| Psi | 60.98% |
| RRE | 50.94% |
| GFP | 46.58% |
| WPRE | 39.71% |

Table S1. Reverse transcription efficiency determination by using *in vitro* synthesized RNA calibrator.


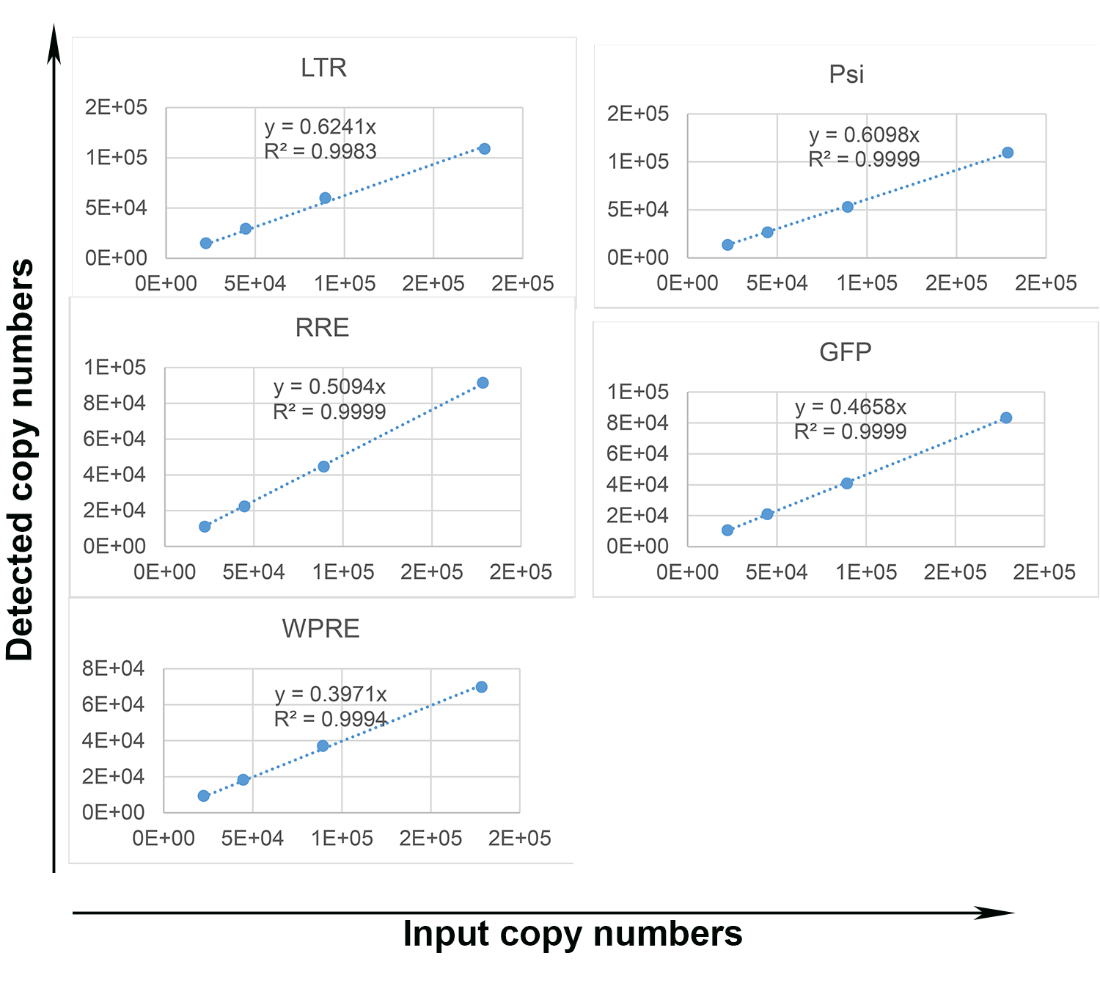


Figure S3. Reverse transcription efficiency determination by using *in vitro* synthesized RNA calibrator.
